# Recent findings on the impact of ErbB receptors status on prognosis and therapy of head and neck squamous cell carcinoma

**DOI:** 10.3389/fmed.2023.1066021

**Published:** 2023-02-02

**Authors:** Camilla Palumbo, Monica Benvenuto, Chiara Focaccetti, Loredana Albonici, Loredana Cifaldi, Alessandra Rufini, Daniela Nardozi, Valentina Angiolini, Arianna Bei, Laura Masuelli, Roberto Bei

**Affiliations:** ^1^Department of Clinical Sciences and Translational Medicine, University of Rome “Tor Vergata”, Rome, Italy; ^2^Saint Camillus International University of Health and Medical Sciences, Rome, Italy; ^3^Academic Department of Pediatrics (DPUO), Ospedale Pediatrico Bambino Gesù, Istituto di Ricovero e Cura a Carattere Scientifico (IRCCS), Rome, Italy; ^4^Department of Biomedicine and Prevention, University of Rome “Tor Vergata”, Rome, Italy; ^5^Department of Experimental Medicine, Sapienza University of Rome, Rome, Italy; ^6^Medical School, University of Rome “Tor Vergata”, Rome, Italy

**Keywords:** head and neck squamous cell carcinoma, EGFR, ErbB receptors, Cetuximab, immune checkpoint inhibitors

## Abstract

Head and neck squamous cell carcinoma (HNSCC) is the sixth most common cancer type, has often an aggressive course and is poorly responsive to current therapeutic approaches, so that 5-year survival rates for patients diagnosed with advanced disease is lower than 50%. The Epidermal Growth Factor Receptor (EGFR) has emerged as an established oncogene in HNSCC. Indeed, although HNSCCs are a heterogeneous group of cancers which differ for histological, molecular and clinical features, EGFR is overexpressed or mutated in a percentage of cases up to about 90%. Moreover, aberrant expression of the other members of the ErbB receptor family, ErbB2, ErbB3 and ErbB4, has also been reported in variable proportions of HNSCCs. Therefore, an increased expression/activity of one or multiple ErbB receptors is found in the vast majority of patients with HNSCC. While aberrant ErbB signaling has long been known to play a critical role in tumor growth, angiogenesis, invasion, metastatization and resistance to therapy, more recent evidence has revealed its impact on other features of cancer cells’ biology, such as the ability to evade antitumor immunity. In this paper we will review recent findings on how ErbB receptors expression and activity, including that associated with non-canonical signaling mechanisms, impacts on prognosis and therapy of HNSCC.

## 1. Introduction

Head and neck (HN) cancers include a heterogeneous group of cancers which differ for histological, molecular and clinical features (1–3). More than 90% of HN cancers are squamous cell carcinomas (HNSCCs) arising from the mucosal epithelium of the oral cavity, pharynx and larynx, while much less common HN subtypes originate from the salivary glands, sinuses, muscles or nerves in the head and neck^1^ (2–4).

HNSCC is the sixth most common cancer type, with a worldwide incidence lately reported to range from about 500,000 to 900,000 cases per year, and with a further 30% increase expected in the next decade (1, 2).

These tumors often have an aggressive course and are poorly responsive to current therapeutic approaches. Indeed, more than half of HNSCC patients is diagnosed with advanced disease for which 5-year survival rates are lower than 50% (4). Moreover, the quality of life of HNSCC patients is often severely compromised as a consequence of both neoplastic growth and multimodality treatments causing pain, impairment of basic functions including eating and speaking, physical disfigurement and psychosocial distress (4).

Epidemiological studies and the definition of risk factors for HNSCC development, have led to the identification of two main subtypes, i.e., Human Papilloma Virus (HPV)-positive and HPV-negative tumors (4). That HNSCC are to be considered different biological entities on the basis of their being associated or not with HPV infection has been further validated by genome sequencing, RNA profiling and clinical data (1–4).

HPV-positive HNSCCs mainly arise in the oropharyngeal region following a latency of 10–30 years from oral infection with high-risk oncogenic HPV strains, primarily HPV-16, and have a more favorable prognosis (3, 4). As for their prevalence, HPV positivity is reported in a percentage of oropharyngeal cancers up to 70% in high income countries (2, 5). Conversely, HPV-negative HNSCCs mainly originate in the oral cavity, hypopharynx and larynx, in most cases in association with longterm tobacco use and alcohol consumption (3, 4).

Onset and progression of HPV-positive and HPV-negative HNSCCs are driven by (partly) different oncogenic pathways. In fact, loss of p53 and pRb tumor suppressor function occurs in both subtypes but as a result of different mechanisms: in HPV-positive tumors these tumor suppressor proteins are inactivated/targeted for degradation by the viral oncoproteins E6 and E7, whereas HPV-negative tumors show frequent mutations in TP53 and CDKN2A genes (~60–80% and ~ 20% of cases, respectively) (1–3). Notably, CDKN2A encodes for the pRb pathway regulator p16INK4A, whose inactivation promotes cell-cycle progression *via* the increased phosphorylation of pRb by cyclin-dependent protein kinases CDK4 and CDK6 (3).

Studies aimed at characterizing the molecular drivers of HNSCCs have identified further differences between HPV-positive and -negative tumors, as reviewed in (1–3), and have also revealed a substantial heterogeneity in the HPV-negative subgroup (3, 6, 7). In this complex landscape, the Epidermal Growth Factor Receptor (EGFR) has emerged as an established oncogene in HNSCC, being overexpressed or mutated in a percentage of cases up to about 90% (2, 7, 8).

**Figure 1:**
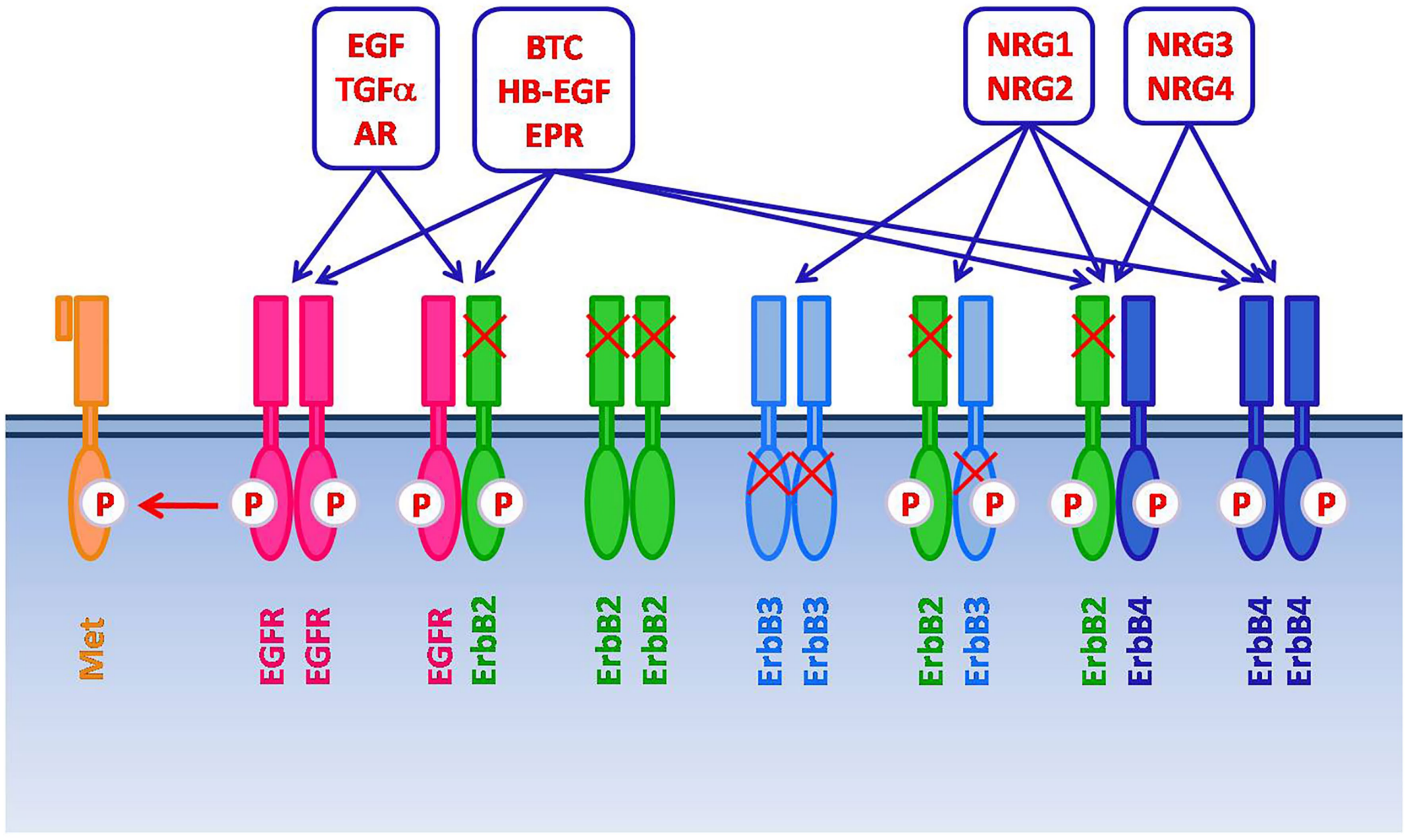
The ErbB/HER family of receptor tyrosine kinases includes four members: EGFR (HER1, ErbB1), ErbB2, ErbB3, and ErbB4 (HER2-4). After the binding with the cognate ligands, ErbB receptors form homo- and heterodimers/oligomers in various combinations. This leads to receptor trans/autophosphorylation and the activation of signaling cascades, resulting in proliferation, differentiation, cell motility, and cell death inhibition. Several ligands have been described, including Epidermal Growth Factor (EGF), Transforming Growth Factor-alpha (TGF-α), Amphiregulin (AR), Betacellulin (BTC), Heparin Binding Epidermal Growth Factor (HB-EGF), Epiregulin (EPR), and Neuregulins (NRGs). The specific ligands for EGFR are EGF, TGF-α and AR. NRG1-2 bind to ErbB3. BTC, HB-EGF, EPR, NRG1-4 bind to ErbB4. BTC, HB-EGF and EPR can also bind to EGFR. ErbB2, which does not have a direct ligand, and ErbB3, which has impaired kinase activity, signal *via* heterodimerization with other members of the family. Moreover, spontaneous dimerization of ErbB2 can occur due to the receptor overexpression. EGFR can also form heterodimers with receptor tyrosine kinases that do not belong to the ErbB family, such as the HGF receptor Met.

The EGFR (HER1, ErbB1) is the prototypical member of the ErbB/HER family of receptor tyrosine kinases, which also includes ErbB2, ErbB3 and ErbB4 (a.k.a. HER2-4) (9). When bound by the cognate ligands, these receptors form homo- and hetero-dimers/oligomers in various combinations. This leads to receptor trans/autophosphorylation and the ensuing activation of interconnected and overlapping signaling cascades (reviewed in 9, 10), resulting in multiple biological responses, including proliferation, differentiation, cell motility, and cell death inhibition (Figure 1).

Beside the seven ligands known to bind the EGFR, which include the Epidermal Growth Factor (EGF) and Transforming Growth Factor α (TGF-α), additional growth factors can lead to EGFR activation *via* its hetero-oligomerization with different ErbB receptor partners (9, 11). In addition, EGFR has been reported to form heterodimers with receptor tyrosine kinases that do not belong to the ErbB family, such as the Hepatocyte Growth Factor (HGF) receptor Met, which also appears frequently overexpressed in HNSCC (1, 3, 11). Such promiscuity partly accounts for the complexity and diversification of the signaling outputs that can be elicited through the EGFR (9). Additional levels of complexity are related to non-canonical mechanisms of EGFR signaling, such as those associated with its nuclear translocation or with the recently reported release of EGFR-containing exosomes from cancer cells (12, 13).

In addition to EGFR, expression of the ErbB2 orphan receptor, which does not have a direct ligand but signals *via* heterodimerization with other members of the family, and of ErbB3 and ErbB4 has also been reported in variable proportions of HNSCCs (6, 14, 15).

While aberrant ErbB signaling has long been known to play a critical role in tumor growth, angiogenesis, invasion, metastatization and resistance to therapy (10), more recent evidence has revealed its impact on other aspects of cancer cells’ biology, such as the ability to evade antitumor immunity (16).

In this paper we will review recent findings on how ErbB receptors expression and activity, including that associated with non-canonical signaling mechanisms, impacts on prognosis and therapy of HNSCC.

## 2. Aberrant expression of ErbB receptors in HNSCC

Aberrant expression of ErbB tyrosine kinase receptors can result from a number of genetic alterations, such as gene amplification, mutation and translocation, and is often associated with aberrant activation of downstream signaling pathways involved in cancer onset and progression. Indeed, ErbB receptors quantitative and qualitative alterations are frequently found in different solid tumors, including HNSCC (2, 17).

When investigating the copy number of ErbB receptors genes in HNSCCs, several studies reported the presence of gene amplification or polysomy. On the other hand, protein expression levels are regarded as a more reliable marker of aberrant receptor activity as compared to quantitative genetic alterations. Indeed, in addition to genetic alterations, several mechanisms may contribute to ErbB protein overexpression, including transcriptional and post-translational mechanisms (17). Relevant studies in this regard, performed in different cohorts of HNSCC patients and published in the last two decades, are reported in Table 1. Overall, the results of these studies indicate that the EGFR protein is overexpressed in 18–90% of patients (median value: 58%), while the gene copy number is increased in 5–55% of patients (median value: 21%) (see Table 1). ErbB2 protein overexpression ranges from 1 to 35% of patients (median value: 7%), with gene copy number alterations found in 2–46% of patients (median value 7%) (see Table 1). Also, ErbB3 and ErbB4 protein are overexpressed in HNSCC patients, with percentages of 21–54% (median value: 43%) and 26% of patients, respectively (see Table 1). Collectively, the available studies clearly indicate that increased expression/activity of one or multiple ErbB receptors is found in the vast majority of patients with HNSCC.

**Table 1:** Aberrant expression of ErbB receptors in HNSCC patients.

Receptor	Protein overexpression	Increased gene copy number	Phosphorylation	Polymorphism / Mutation	References
EGFR	18–30%				(18)
	33%				(19)
	34%				(20)
	38%				(21)
	39%	45%			(22)
	45%				(23)
	45%			4% exon 18–21 mutation	(24)
				17–32% Q787Q polymorphism	
	46–85%				(25)
	47%				(26)
	47%	31%			(27)
	48%	14%			(28)
	49%				(29)
	49%				(30)
	50%	17%			(31)
	56%				(32)
	57%				(33)
	57%		29% at Tyr 1173		(34)
			20% at Tyr1068, 1086 and 1114		
	57–60%				(35)
	58%	13%			(36)
	63–85%	5–12%			(37)
	65%				(38)
	71–76%				(39)
	72%				(40)
	73%				(41)
	73%				(42)
	73%			17% EGFRvIII	(43)
	75%		20% at Tyr1068		(44)
			47% at Tyr1148		
	76%	8%			(45)
	77%	26%		14.5% exon 19–20 mutation	(46)
	81%				(47)
	82%	36%			(48)
	86%				(48)
	90%	21%		1% mutated	(49)
		11%			(50)
		16%			(51)
		20%			(52)
		23–32%		18% EGFRvIII	(53)
		50%		33–38% EGFRvIII	(54)
		55%		21% EGFRvIII	(55)	
			38% at Tyr1068	5% exon 20 mutation	(56)
				7% exon 20 mutation	(57)
				9% EGFRvIII	(58)
				7% exon 19 mutation	(59)
				21% EGFRvIII	
				56% EGFR-K_521_	(60)
ErbB2	1%				(30)
	2%	2%			(49)
	1–3%	3–5%			(61)
	11–19%				(62)
	29%				(26)
	35%	12%			(63)
		9.50%			(64)
		46.50%			(65)
ErbB3	21%				(26)
	27%				(66)
	43%				(67)
	50–54%				(68)
ErbB4	26%				(26)
	26%				(67)
				1% mutated	(49)

Conversely, it has been reported that the expression of *EGFR* can be negatively regulated by the methylation of its promoter, which is more often associated with HPV infection (24). In this regard, it is of note that HPV-positive HNSCCs have a tendency to express lower levels of EGFR as compared to HPV-negative tumors (29, 52, 69, 70). On the other hand, as regards the impact of HPV status on the expression levels of ErbB receptors, HPV-positive HNSCC have been found to express higher levels of ErbB2, ErbB3 and ErbB2:ErbB3 heterodimers as compared to HPV-negative tumors (70). This finding is remarkable, since it suggests that patients with HPV-positive HNSCC may benefit from therapeutic regimens based on agents able to target simultaneously multiple ErbB receptors.

Clearly, the evaluation of the phosphorylated receptors levels would represent an even better biomarker of their activity as compared to the mere expression levels and, accordingly, different authors argue against the predictive value of ErbB protein amounts in tumor tissues in the absence of data on their tyrosine kinase activity (34). Furthermore, beside the reported alterations in expression levels, ErbB activity is known to be affected by polymorphisms and mutations in the encoding genes. In this regard, different authors have reported that, albeit at an overall low frequency, EGFR mutations affect a proportion of HNSCCs (24, 56, 57, 59). These mutations frequently occur in the intracellular kinase domain (ICD), in particular in exons 18–21, often leading to aberrant receptor signaling and conferring resistance to targeted therapies (8; Table 1). As for their frequency, a study reported the presence of EGFR ICD mutations in 57% of samples from a cohort of Saudi HNSCC patients (71). However, in most studies EGFR ICD mutations have been found in a percentage of cases lower than 10% (72; Table 1). Regarding the mutational status of the EGFR extracellular domain (ECD), this has been investigated in a limited number of studies in HNSCC, in spite of its potential therapeutic implications (73). In fact, the ECD mutations G33S, N56K and G465R have been found in HNSCC cells and reported to prevent Cetuximab binding to the receptor (73, 74). A larger number of studies has instead focused on the expression of EGFRvIII, a mutant EGFR with in-frame deletion of exons 2–7, which is incapable of binding ligands due to the lack of a portion of the ligand-binding domain, but is characterized by low levels of constitutive activity (54). The expression of EGFRvIII in HNSCC cells has been linked to enhanced growth and resistance to both cisplatin and agents targeting wild-type EGFR (75). However, while the expression of this mutant has been found in approximately 20% of HNSCC patients by different authors (54, 55), conflicting results obtained in other studies have raised doubts on its clinical relevance in HNSCC (6, 58).

Polymorphic variants of the EGFR are also known to differ for expression levels, function and sensitivity to targeted agents (17, 76). The EGFR-K_521_ (K-allele), resulting from a single nucleotide polymorphism which involves the EGFR ECD, has been found in 56% of HNSCC patients and shown to display a reduced affinity for Cetuximab (60). At the opposite, the EGFR Q787Q synonymous polymorphism appears to confer greater sensitivity to EGFR tyrosine kinase inhibitors (TKIs), due to a long noncoding RNA-mediated mechanisms, and has been reported in 17% of HPV-related and 32% of HPV-unrelated oropharyngeal HNSCC patients (24).

## 3. ErbB receptors and prognosis of HNSCC

Studies aimed at correlating ErbB protein expression levels and prognosis of HNSCC have reported variable results. Multiple factors are responsible for this inherent variability, including differences in tumor sites, use of different antibodies, detection techniques, immunostaining scoring systems and also the different evaluation of the receptors subcellular distribution (17, 62). Nonetheless, there is a rather general consensus on the correlation between EGFR expression levels, poor prognosis and worse treatment outcomes in patients with HNSCC, while there is no definitive evidence that *EGFR* gene copy number and mutations may have prognostic value in this group of cancers (1, 2, 8, 17, 33). In particular, EGFR overexpression has been associated with radiotherapy resistance, loco-regional treatment failure, higher rates of metastatization, and with reduced disease-free, progression-free and overall survival in different cohorts of patients (8, 17, 77, 78). Worthy of note, several authors remarked that the significance of the observed correlations is critically dependent on the assessment of EGFR protein levels in cancer tissues by means of quantitative image analysis and scoring systems, taking into account both intensity and extent of the immunostaining (17, 78). Even though HPV-positive HNSCCs have a tendency to express lower levels of EGFR, according to some authors this receptor holds prognostic value also in this subgroup of tumors. In fact, it has been reported that the outcome of HPV-positive tumors with higher EGFR levels is worse as compared to that of HPV-positive tumors with low EGFR (29, 52, 69). However, others reported that the impact of EGFR on outcome may be limited to HPV-negative HNSCCs (32, 79).

It should also be also considered that an aberrant EGFR signaling may result from additional mechanisms beside its overexpression or mutation, such as transactivation by different receptors, including for instance the already mentioned Met receptor, or increased expression of the cognate ligands (3, 11, 17). Consistent with this consideration, in a study performed on HNSCC cell lines and tumor specimens it was observed that EGFR expression levels were not correlated with EGFR activity, evaluated *via* its phosphorylation status at multiple tyrosine residues (34). Accordingly, rather than EGFR protein levels, phosphorylated receptor levels should represent a better biomarker for EGFR pathway activation in tumor samples (80). However, the levels of activated EGFR in HNSCC have been investigated in a limited number of studies, with partly conflicting results regarding the impact of receptor activity on clinical outcome (44, 53).

While HER2 amplification/overexpression is a marker of poor prognosis in different cancers, including breast, ovary and lung carcinomas, the prognostic value of this receptor in HNSCC is still a matter of debate (61, 62, 81). By the way, even though ErbB2 is overexpressed in a fraction of HNSCCs, its expression levels appear on the whole lower in this type as compared to other types of cancer (62). A recently published study highlights how the association between ErbB2 overexpression and clinical outcome can be dependent on the use of different systems for scoring the receptor levels in HNSCC tissue samples (62). In this study the authors investigated ErbB2 expression by immunohistochemistry in 120 HNSCC tissue sections including laryngeal, oral cavity and oropharyngeal squamous cell carcinomas, and evaluated ErbB2 immunostaining using two systems: the conventional scoring system approved by the FDA, which takes into account the degree of membrane staining in >10% of cells, and an H-score-based system, in which an H-score value is obtained for each section by multiplying the intensity score by a proportion score based on the percentage of stained cells. According to both scoring systems, the majority of ErbB2-positive tumors were poorly differentiated, stage IV tumors with lymph nodal involvement. However, a different percentage of ErbB2-positive tumors was obtained using the H-score system as compared to the conventional system (19% vs. 11%, respectively), and ErbB2 overexpression was associated with decreased overall survival when evaluated by H-score only. In particular, median survival was 11 months for ErbB2-positive patients and 49 months for ErbB2-negative patients by H-score. In the same study it was evaluated the association between ErbB2 levels and clinical outcome based on data downloaded from The Cancer Proteome Atlas^2^ and it was found that ErbB2 protein expression had no effect on survival of patients with oral and oropharyngeal squamous cell carcinoma, while it was associated with improved survival in patients with laryngeal HNSCC. These results, coupled with the conflicting findings obtained in other studies, indicate that it is unlikely that ErbB2 could be a useful prognostic marker for HNSCC (61, 62, 81–83).

As compared with EGFR and ErbB2, a smaller number of studies have investigated the association between the expression of ErbB3 and ErbB4 and prognosis of HNSCC. As regards ErbB3, different authors agree on its value as a predictor of poor clinical outcome in HNSCC. In a study performed on a large cohort of HNSCC patients, membranous ErbB3 overexpression was associated with worse overall survival and was significantly increased in metastatic lesions as compared to primary tumors (84). In different cohorts of HNSCC patients, ErbB3 expression levels have also been found to correlate with nodal stage, poor relapse-free, disease-free, and overall survival (68, 85, 86).

Still, a more complex scenario emerges from a recent study, where the subcellular distribution of ErbB3 in laryngeal HNSCC cells was taken into account (87). Indeed, ErbB3, as well as ErbB4, appears to play different roles in HNSCC progression depending on its membranous/cytoplasmic vs. nuclear localization (87, 88). This aspect, which will be discussed in the next paragraph in the context of non-canonical mechanisms of ErbB signaling, can also be partly responsible for the discordant data regarding ErbB4 as prognostic marker for HNSCC. Actually, only sparse data are available on the impact of ErbB4 status on HNSCC, and the discordant results in this regard may also be ascribed to the different site of origin of the tumors investigated. In fact, a correlation has been found by different authors between ErbB4 membranous/cytoplasmic expression, lymph node metastasis and risk of recurrence in oral HNSCC (89, 90). Conversely, ErbB4 overexpression has been reported as a favorable prognostic factor in tongue and, in association with its nuclear localization, in laryngeal HNSCC (85, 88, 91).

On the other hand, many lines of evidence indicate that, beside the expression of individual ErbB receptors, the simultaneous expression of multiple ErbB family members can be a stronger predictor for outcome of HNSCC (92, 93). Indeed, co-expression of different ErbBs allows receptor heterodimerization, which in turn activates cooperative and diversified downstream signaling cascades (9, 10). In an early report, the expression of each ErbB family member was significantly associated with shortened survival in patients with oral HNSCC, but the co-expression of EGFR, ErbB2 and ErbB3 had an improved predicting power (93). In another study aimed at investigating the relationship between clinical parameters and single versus paired overexpression of ErbB family members in patients with oral HNSCC, overexpression of ErbB1 and ErbB4 was associated with a lower survival, but the simultaneous overexpression of both receptors predicted the worst overall and disease-free survivals (90). Consistent with these findings, in a previous study by our group performed on oral cavity epithelium samples, including invasive and *in situ* carcinomas, benign lesions and normal mucosa, the simultaneous expression of three or four ErbB receptors was correlated with tumor invasion (94). Finally, in a recent paper the prognostic stratification of patients with EGFR-positive advanced laryngeal squamous cell carcinoma was improved by taking into account the simultaneous expression of nuclear ErbB3 (as further detailed in the next paragraph) (87). On the whole, these findings indicate that cooperative signaling by ErbB receptors plays a significant role in the pathogenesis and outcome of HNSCC, which deserves further investigation.

## 4. Involvement of non-canonical ErbB signaling in HNSCC

In addition to receptor overexpression or mutation, more recent findings point to non-canonical mechanisms of ErbB signaling as important players in different cancers (12, 95). Non-canonical ErbB signaling involves for instance mechanisms mediated by different subcellular localizations of the receptors, and most studies in this respect have been focused on nuclear EGFR (12, 95).

**Figure 2:**
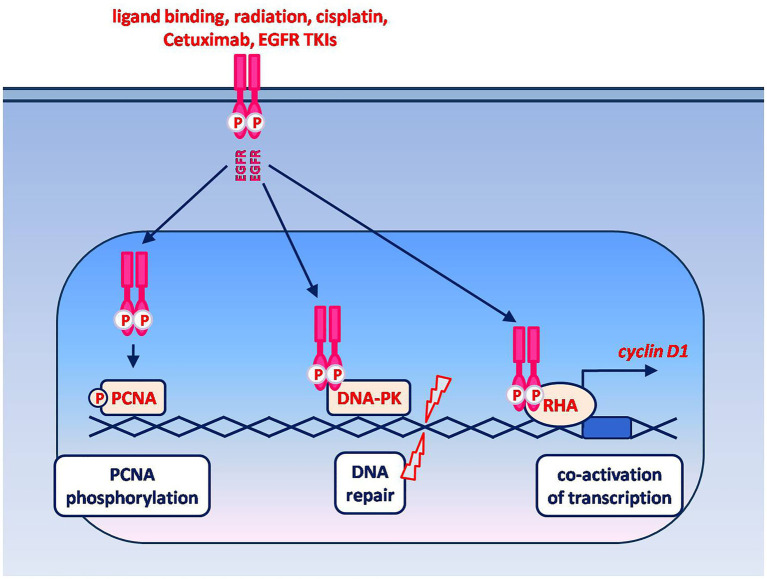
Non-canonical signaling by nuclear EGFR. EGFR nuclear translocation is known to be induced by ligand binding as well as by cell exposure to radiations, drugs and EGFR-targeting agents. The known oncogenic functions of nuclear EGFR include: phosphorylation and stabilization of chromatin-bound PCNA, which in turn plays an essential role in DNA replication and repair; promotion of DNA repair *via* protein–protein interaction with the DNA-dependent protein kinase (DNA-PK); co-activation of transcription *via* the interaction with different transcriptional regulators. The interaction between nuclear EGFR and RNA helicase A (RHA) leads to an enhanced expression of cyclin D1. Among the other genes whose expression is enhanced by nuclear EGFR, in association with various transcription factors, are those encoding for c-Myc, B-Myb, Aurora Kinase A and thymidylate synthase.

Different stimuli, that include (but are not limited to) ligand-binding, can induce EGFR nuclear translocation (Figure 2). Once in the nucleus, one of the functions of EGFR is to promote cell proliferation by phosphorylating and stabilizing the proliferating cell nuclear antigen (PCNA). Accordingly, nuclear EGFR is typically found in rapidly dividing cells (95). In addition, nuclear EGFR can promote DNA damage repair and act as a co-transcription factor leading to the increased expression of oncogenes (e.g., Cyclin D1, c-Myc, B-Myb, Aurora Kinase A). Remarkably, these latter functions are mediated by protein–protein interactions and do not appear to require the receptor tyrosine kinase activity (95). This finding and the subcellular localization of nuclear EGFR suggest that its expression could predict clinical resistance to both EGFR-targeted therapeutic antibodies and TKIs (95).

A correlation between nuclear EGFR expression, poor survival and chemo-radiation resistance has been in fact demonstrated in various types of cancer, including HNSCC (95). In particular, nuclear EGFR appears associated with induced chemo-radiation resistance, since its level was found to increase following *in vitro* irradiation, cisplatin exposure, or treatment of HNSCC cells with Cetuximab or with the EGFR TKIs Erlotinib and Lapatinib (12, 96–98). Moreover, an association was found between increased sensitivity of cultured HNSCC cells to the cytotoxic effects of irradiation, cisplatin and Cetuximab, and reduced levels of EGFR nuclear translocation (96, 98).

A correlation between nuclear EGFR expression and HNSCC clinical parameters has also been reported by several authors. In a study on laryngeal HNSCC, a higher frequency of strong nuclear EGFR was found in invasive tumors compared to laryngeal dysplasia and vocal cord polyps, and high nuclear EGFR expression levels correlated with worse overall cancer patients’ survival (39). In oropharyngeal HNSCC high nuclear EGFR expression levels were associated with reduced responses to radiation therapy, higher risk of local recurrence and lower overall survival (99, 100). Finally, in a study on the expression of total and nuclear EGFR in relation to the HPV infection surrogate marker p16, it was shown that both total and nuclear EGFR levels were higher in p16-negative tumors compared to p16-positive tumors (69). On the whole, the reported findings indicate that EGFR nuclear localization is a negative prognostic factor in HNSCC and suggest that it may be regarded as a biomarker for clinical resistance as well as a potential therapeutic target (95).

Worthy of note, also the other ErbB receptors are known to translocate to the nucleus and play a role in transcriptional regulation (95, 101). However, unlike nuclear EGFR, localization of ErbB3 and ErbB4 in the nucleus of HNSCC cells appears associated with a more favorable prognosis. Evidence in this respect comes from studies on laryngeal HNSCC in which ErbB3 was primarily observed in the nuclear compartment of tumor cells in association with variable cytoplasmic staining, and low expression levels of the receptor were associated with high proliferative indices, and with shorter relapse-free and overall survival (88, 91). Further, EGFR-positive laryngeal tumors co-expressing nuclear ErbB3 had a better prognosis as compared to those that expressed EGFR without ErbB3 or in association with cytoplasmic ErbB3 (87). Moreover, based on the observation that ErbB3 was never expressed alone, but always co-expressed with ErbB2, both in the presence and absence of EGFR, the authors of these studies speculated that ErbB3 nuclear localization may play a favorable role by preventing the formation of ErbB heterodimers at the cell membrane and the ensuing activation of pro-tumoral downstream signaling pathways (87).

As for ErbB4, this receptor is known to undergo ligand-induced intramembrane-regulated proteolysis (RIP) leading to the nuclear translocation of its cytoplasmic domain (101, 102). In a study by our group comparing the expression and distribution of ErbB receptors between HNSCCs and adjacent normal mucosa, ErbB4 was overexpressed in ~26% of the investigated carcinomas and, in addition to cytoplasmic staining, a strong nuclear ErbB4 immunostaining was observed in ~18% of carcinomas (26). Such nuclear localization of ErbB4 was found in tumors graded from 1 to 3, according to WHO classification, thus appearing unrelated to the degree of tumor differentiation (26).

More recently, both nuclear and cytoplasmic positivity for ErbB4 has also been reported in a proportion of laryngeal HNSCCs (~43%, 67 cases) and a more prominent localization of ErbB4 in the nuclear compartment was associated with longer relapse-free and overall survival (88, 91). This finding is consistent with the reported role of nuclear ErbB4 in mediating growth arrest or apoptosis in various tissues, even though its impact on the biological and clinical behavior of different cancer types remains controversial (102, 103).

Recently, the multifaceted function of tumor cell-derived exosomes has emerged as an important field in cancer research, and in this regard novel, non-canonical mechanisms of action have been reported for both the EGFR and ErbB2. Exosomes, i.e., nanovesicles released by cells in the interstitial space and found in various bodily fluids, such as serum, plasma, saliva, urine, etc., act as mediators of intercellular communication by delivering a complex cargo of sorted proteins, lipids, nucleic acids and metabolites from donor to recipient cells (104). A growing number of studies is demonstrating that the cargo of tumor-derived exosomes is specifically enriched in signaling molecules able to promote cancer progression and remodeling of the tumor microenvironment (104), and different cancer types have been reported to release EGFR- or ErbB2-containing exosomes (13, 95).

Indeed, exosomal transfer of tumor-derived EGFR to both local and distant recipient cells has been found to promote cancer cell growth, metastases and drug resistance as well as angiogenesis, metastatic niche formation, and suppression of anti-tumor immunity (13, 95, 105). Although reported in a more limited number of studies, similar findings have been published as regards the exosomal transfer of tumor-derived ErbB2 (95, 106, 107).

As in other cancer types, exosomes are deeply involved in HNSCC progression, and the correlation between exosome release and HNSCC aggressiveness is supported by multiple lines of evidence (108, 109). In this context, the release of EGFR-containing exosomes from HNSCCs has been demonstrated to occur both *in vitro* and *in vivo*. Indeed, HNSCC cultured cells have been reported to release both EGFR- and phospho-EGFR containing exosomes (110, 111). In addition, tumor-derived exosomes immunocaptured from HNSCC patients’ plasma have been found to contain high amounts of immunosuppressive and tumor growth-promoting mediators such as PD-L1, FasL, TGF-β and EGFR. Remarkably, the exosomal levels of these proteins, including the EGFR, correlated with clinicopathological parameters, being higher in patients with stage III/IV disease and lymph node metastases versus those with stage I/II disease and without lymph node metastases (112).

Fujiwara and colleagues showed that in Cetuximab-treated oral HNSCC cells the therapeutic antibody was secreted within EGFR-containing vesicles (including exosomes and microvesicles), thereby providing evidence of a mechanism by which the release of EGFR-containing vesicles may reduce the antibody therapeutic efficacy (110). Further, they demonstrated that the release of EGFR-containing vesicles by oral squamous cell carcinoma was increased by EGF stimulation and was able to drive carcinogenic epithelial-mesenchymal transition in recipient immortalized oral epithelial cells (113).

On the whole, these findings highlight that more research is warranted to fully define the involvement of ErbB receptors-containing exosomes in HNSCC, to validate their possible relevance as non-invasive diagnostic/prognostic markers and to explore their potential as therapeutic targets (13, 108, 109).

## 5. ErbB receptors and therapy of HNSCC

Early-stage HNSCCs (30–40%) are currently managed with single modality approaches (mainly surgical resection for oral cavity cancers and radiation for larynx and pharynx cancers), with long-term survival rates achieved in about 70–90% of patients (2, 4). However, the majority of patients are diagnosed with advanced disease, requiring multimodality interventions. These usually consist of surgery followed by radiation with or without (platinum-based) chemotherapy for cancers of the oral cavity, and primary chemoradiation for cancers arising in the pharynx or larynx (2, 4, 5). Beside their severe impact on quality of life, such interventions have limited effects, recurrent/metastatic disease affects more than 65% of patients, and 5-year survival rates following a diagnosis of advanced HNSCC are lower than 50% (1, 4).

Due to the growing body of evidence on the involvement of EGFR in HNSCC, many preclinical and clinical study have investigated the therapeutic potential of EGFR-targeting agents in this group of tumors. These studies have led to the approval of Cetuximab in combination with radiation therapy (with or without chemotherapy) for patients with locally advanced HNSCC (2, 114). Cetuximab, a chimeric monoclonal antibody that competes with natural ligands for binding to the extracellular region of EGFR, was approved for HNSCC by the European Medicines Agency (EMA) in 2004 and by the Food and Drug Administration (FDA) in 2006, remaining the only approved targeted agent for HNSCC until the recent introduction of immune checkpoint inhibitors (ICIs) (1, 2, 114). Indeed, HNSCCs, and in particular HPV-negative tumors, are highly radioresistant, and Cetuximab was proven to act as a radiosensitizer able to improve loco-regional control as well as to increase overall survival from 29.3 to 49.0 months, when combined with radiation alone (1, 2, 114, 115). Over time, however, resistance to both radiotherapy and Cetuximab develops in the majority of HNSCC patients, hence allowing for relapse and disease progression (1, 114, 115). The mechanisms responsible for Cetuximab resistance are multifactorial and appear to involve, among the others, the increased expression/activity of different receptor tyrosine kinases (including other ErbB family members and Met) or of the same EGFR, increased production of EGFR ligands, increased EGFR subcellular localization in the nuclear compartment (12, 97, 114, 116). Due to the involvement of Met receptor in Cetuximab resistance, many studies have investigated the dual targeting of Met and EGFR as a therapeutic strategy for HNSCC. However, although preclinical data support the co-targeting of EGFR and Met in these tumors (117), according to the recently published results of a phase II trial performed in patients with recurrent/metastatic HNSCC, the combination between Cetuximab and the Met inhibitor Tivantinib did not improve response rates or patients survival compared with Cetuximab alone, while it was associated with increased toxicities (118–120).

In addition to Cetuximab, therapeutic regimens including different ErbB-targeting agents have been and are being investigated in HNSCC patients (115). However, the results of the clinical studies published so far show that many among these agents have an overall modest clinical efficacy, that does not appear on the whole superior to that of Cetuximab (1, 115). In this regard, a modest efficacy in terms of clinical outcomes has been reported for the fully human anti-EGFR antibodies Panitumumab and Zalutumumab, and for Duligotuzumab, a dual-action antibody directed against EGFR and ErbB3 (115). Among the anti-EGFR therapeutic antibodies, some encouraging results have instead been obtained with Nimotuzumab, whose binding requires that EGFR is expressed at high density on the surface of the target cells, thus resulting in selective activity on EGFR-overexpressing tumor cells as compared to normal cells (115, 121). As for ErbB receptors TKIs, Gefitinib and Erlotinib, which act as reversible inhibitors of EGFR, and Lapatinib, a reversible inhibitor of both EGFR and ErbB2, have also shown a modest activity in HNSCC patients (1, 115). Conversely, Afatinib, a multi-targeted and irreversible TKI targeting EGFR, ErbB2 and ErbB4, has shown clinical benefits in different cohorts of HNSCC patients, comparable or superior to that of Cetuximab (1, 115). A list of selected, recently completed, recruiting and active trials using ErbB-targeted agents, alone or in combination with different treatments, is reported in Table 2.

**Table 2:** Clinical trials with ErbB receptors-targeting drugs in HNSCC patients.

ErbB receptors targeting drug	ErbB receptor target	Combination drug	ClinicalTrial.gov identifier	Phase	Status
Anti-ErbB receptors antibodies					
Cetuximab	EGFR	–	NCT03769311	II	Completed
Cetuximab or Imgatuzumab	EGFR	–	NCT01046266	I	Completed (122)
Cetuximab	EGFR	Paclitaxel, Cisplatin	NCT00933387	II	Completed
Cetuximab	EGFR	Paclitaxel, Carboplatin, radiotherapy	NCT00343083	II	Completed
Cetuximab	EGFR	nab-Paclitaxel, Cisplatin, radiotherapy	NCT02573493	II	Active, not recruiting (123)
Cetuximab	EGFR	Ficlatuzumab (anti-HGF antibody)	NCT02277197	I	Completed
Cetuximab	EGFR	EGFR antisense DNA, radiotherapy	NCT00903461	I	Terminated
Cetuximab	EGFR	FATE-NK100 (donor-derived NK cells)	NCT03319459	I	Completed
Cetuximab	EGFR	Durvalumab (anti-PD-L1 antibody), radiotherapy	NCT03051906	I-II	Terminated (124)
Cetuximab	EGFR	Ipilimumab (anti CTLA-4 antibody), radiotherapy	NCT01935921	I	Active, not recruiting (125)
Cetuximab	EGFR	Pembrolizumab (anti-PD-1 antibody)	NCT03082534	II	Active, not recruiting (126)
Cetuximab	EGFR	Avelumab (anti-PD-L1 antibody)	NCT03494322	II	Active, not recruiting
Cetuximab	EGFR	Avelumab (anti-PD-L1 antibody), radiotherapy	NCT02999087	III	Active, not recruiting (127)
Panitumumab	EGFR	Carboplatin, Cisplatin, Docetaxel, Paclitaxel, Fluorouracil, radiotherapy	NCT00513383	I	Completed
Panitumumab	EGFR	Cisplatin	NCT00547157	II	Completed (128)
Panitumumab	EGFR	Cisplatin, Fluorouracil	NCT00460265	III	Completed (129)
Zalutumumab	EGFR	–	NCT01054625	I-II	Completed
Zalutumumab	EGFR	Radiotherapy	NCT00707655	I-II	Terminated
Zalutumumab	EGFR	Radiotherapy	NCT00496652	III	Completed
Nimotuzumab	EGFR	Cisplatin, Fluorouracil, Docetaxel	NCT01425736	II	Completed
Sym004	EGFR	–	NCT01417936	II	Completed (130)
ABBV-221	EGFR	–	NCT02365662	I	Terminated
ABBV-321	EGFR	–	NCT03234712	I	Completed
A166	ErbB2	–	NCT03602079	I-II	Active, not recruiting
Margetuximab	ErbB2	Tebotelimab^1^ (PD-1-targeting bispecific protein)	NCT03219268	I	Active, not recruiting
BDC-1001	ErbB2	Nivolumab (anti-PD-1 antibody)	NCT04278144	I-II	Recruiting
SBT6050	ErbB2	Pembrolizumab, Cemiplimab (anti-PD-1 antibodies)	NCT04460456	I	Active, not recruiting
LJM716	ErbB3	–	NCT01598077	I	Completed (131)
MCLA-128	ErbB2	–	NCT04100694		Available
	ErbB3				
ErbB receptors tyrosine kinase inhibitors					
Erlotinib	EGFR	Docetaxel, radiotherapy	NCT00049283	I	Completed
Erlotinib	EGFR	Docetaxel, radiotherapy	NCT00720304	II	Completed
Erlotinib	EGFR	Bevacizumab (anti- VEGF antibody), Sulindac	NCT00392665	II	Terminated
Erlotinib	EGFR	Temsirolimus (Mammalian Target of Rapamycin (mTOR) inhibitor)	NCT01009203	II	Terminated
Gefitinib	EGFR	Paclitaxel, radiotherapy	NCT00083057	I	Completed
Zactima	EGFR	Docetaxel	NCT00459043	II	Completed
Lapatinib	EGFR, ErbB2	–	NCT00371566	II	Completed
Lapatinib	EGFR, ErbB2	–	NCT00098631	II	Completed
Lapatinib	EGFR, ErbB2	Carboplatin, Cisplatin, Docetaxel, Fluorouracil	NCT00498953	I-II	Completed
Lapatinib	EGFR, ErbB2	Cisplatin, radiotherapy	NCT00387127	II	Completed (132)
Lapatinib	EGFR, ErbB2	Cisplatin, radiotherapy	NCT01711658	II	Active, not recruiting
Lapatinib	EGFR, ErbB2	Cisplatin, radiotherapy	NCT00424255	III	Completed (133)
Lapatinib and Catuximab	EGFR, ErbB2	–	NCT01184482	I	Completed
CUDC-101	EGFR, ErbB2	–	NCT01171924	I	Completed
CUDC-101	EGFR, ErbB2	Cisplatin, radiotherapy	NCT01384799	I	Completed
Afatinib	EGFR, ErbB2, ErbB4	Pembrolizumab (anti-PD-1 antibody)	NCT03695510	II	Completed (134)
Dacomitinib	EGFR, ErbB2, ErbB4	–	NCT01449201	II	Completed
Dacomitinib	EGFR, ErbB2, ErbB4	Cisplatin, radiotherapy	NCT01737008	I	Completed (135)
ErbB receptors-targeted Chimeric Antigen Receptor (CAR) immune cells					
T1E28z CAR-T cells	ErbB dimers	–	NCT01818323	I	Recruiting (136)
CT-0508	ErbB2	–	NCT04660929	I	Recruiting
ACE1702	ErbB2	Cyclophosphamide, Fludarabine	NCT04319757	I	Recruiting
CAR T cells	ErbB2	CAdVEC oncolytic adenovirus	NCT03740256	I	Recruiting (137)
ErbB receptor-targeted antisense therapy					
BB-401	EGFR	–	NCT03433027	II	Completed

*Anti-ErbB receptors antibodies*: A166, anti-ErbB2 antibody conjugated with auristatin; ABBV-221 and ABBV-321, antibody-drug conjugates targeting EGFR; BDC-1001, ErbB2-targeting TLR7/8 immune-stimulating antibody conjugate (ISAC); Cetuximab, anti-EGFR antibody; Imgatuzumab, glycoengineered anti-EGFR antibody; LJM716, anti-ErbB3 antibody; Margetuximab, anti-ErbB2 antibody; MCLA-128, anti-ErbB2/ErbB3 antibody; Nimotuzumab, anti-EGFR antibody; Panitumumab, anti-EGFR antibody; SBT6050, anti-ErbB2 antibody conjugated with Toll-Like-Receptor 8 agonist; Sym004, anti-EGFR antibody mixture; Trastuzumab, anti-ErbB2 antibody; Zalutumumab, anti-EGFR antibody. *ErbB receptors tyrosine kinase inhibitors*: Afatinib, EGFR/ErbB2/ErbB4 inhibitor; CUDC-101, triple inhibitor of EGFR, ErbB2 and histone deacetylase; Dacomitinib, EGFR/ErbB2/ErbB4 inhibitor; Erlotinib, EGFR inhibitor; Gefitinib, EGFR inhibitor; Lapatinib, EGFR/ErbB2 inhibitor; Zactima, dual inhibitor of EGFR and Vascular Endothelial Growth Factor (VEGF) receptor-2. *ErbB-targeted Chimeric Antigen Receptor (CAR) cells*: T1E28z CAR-T cells, T cells engineered to target ErbB dimers; ACE1702, anti-ErbB2 CAR NK cells; CT-0508, anti-ErbB2 CAR macrophages. *ErbB-targeted antisense therapy*: BB-401, EGFR antisense DNA. ^1^Tebotelimab: a bispecific DART protein targeting PD-1 and the immune checkpoint Lymphocyte-activation gene 3 (LAG-3).

## 6. ErbB receptors and immunotherapy of HNSCC

In 2016 Immune Checkpoint Inhibitors (ICIs) entered the clinical practice for HNSCC with the approval of the anti-Programmed cell Death protein 1 (PD-1) antibodies Nivolumab and Pembrolizumab for patients with recurrent or metastatic tumors (5). Indeed, promising results were obtained in clinical trials based on these agents, both in terms of improved survival and limited treatment-related toxicities (1, 2, 5).

The PD-1/PD-L1 checkpoint is a central mediator of immunosuppression in the tumor immune microenvironment. The PD-1 receptor is expressed, among the others, on the surface of T cells, and its binding to PD-L1 ligands on the surface of tumor cells (and other cells in the tumor microenvironment) negatively regulates T cells functions and allows tumor evasion of immune surveillance (138). By blocking the PD-1/PD-L1 interaction, ICIs can release T cell inhibition, thus restoring antitumor immunity (138). Preclinical data also suggest that the combined blockade of PD-1 and Cytotoxic T Lymphocyte Antigen-4 (CTLA-4) immune checkpoints could be beneficial in HN cancers (139). In this context, the evidence that ErbB receptors have important immune-modulatory effects has provided the rational basis for combining ICIs and ErbB-targeting agents in HNSCC patients.

Indeed, abnormal signals by ErbB family members, in addition to playing an essential role in tumorigenesis, are accountable for the evasion of antitumor immunity in many cancers, including HNSCC (16). Although the mechanisms are still incompletely understood, inhibitory effects on the adaptive antitumor immune response are associated with the creation of a tumor microenvironment conditioned by many factors affected by aberrant ErbB receptor signaling. In HNSCC the main cellular components of the tumor microenvironment are T lymphocytes, tumor-associated macrophages (TAMs), myeloid-derived suppressor cells (MDSCs), natural killer cells (NKs), and cancer-associated fibroblasts (CAF) (140). Several cell types in the microenvironment of HNSCC tumors, including regulatory T cells (Tregs), CAFs, and TAMs have been shown to mediate immunosuppression and dysfunction of antitumor immunity (141). In fact, HNSCCs, in particular those of the inflamed/mesenchymal subtype (6, 142), often have a high infiltrate of CD8^+^ T cells, which, however, is not always linked to a favorable prognosis (143). Indeed, although higher numbers of CD8^+^ T lymphocytes have been correlated with improved outcomes of HNSCC by some authors (144–146), often T cells present in HNSCC microenvironment are dysfunctional or “exhausted.” In this regard, high numbers of immunosuppressive Tregs are found among tumor infiltrating lymphocytes in HNSCC, and their presence has been associated with unfavorable prognosis and resistance to radiotherapy (147).

Actually, several findings support a role for ErbB receptors in mediating tumor immune escape. Oncogenic signals *via* EGFR and ErbB2 can induce the overexpression of PD-L1 and the production of immunosuppressive cytokines including transforming growth factor beta (TGF-β), vascular endothelial growth factor (VEGF) or IL-10 (148, 149). Furthermore, EGFR signaling can participate to the suppression of immune responses *via* recruitment and activation of Tregs as well as through the reduction of T cell chemo-attractants (149). Moreover, both EGFR and ErbB2 have been reported to downregulate HLA class-I mediated peptide presentation (148). Further, aberrant ErbB signaling has been associated with the decrease of Th1 response, the induction of the exhaustive phenotype of CD8^+^ T lymphocytes and the immune cells switch from a pro-inflammatory to a pro-tumor phenotype (143).

The generation of abnormal proteins, which have not been previously recognized by the immune system (neoantigens), derived by HNSCC cells with inherent genetic instability could trigger CD8^+^ T cell responses and contribute to the elimination of cancer cells (150). In this regard, the lack or reduced presentation of neoantigens *via* MHCI or MHCII can adversely affect the adaptive antitumor immune response. Conversely, the presentation of neoantigens *via* MHC molecules can have a significant impact on clinical responses to immunotherapies, such as those targeting immune checkpoints mediated by CTLA-4 signaling and the PD-1/PD-L1 axis (151). In this context, aberrant ErbB signaling can also alter the tumor cells’ transcriptome, including the expression of tumor-associated antigens (TAA) and tumor-specific antigens (TSA), therefore influencing both the pool of processing peptides available for antigen presentation and the MHC repertoire present at the surface of tumor cells (152).

Interesting results have also been reported on the link between ErbB receptors expression and spontaneous immune responses in HNSCC. Different studies have demonstrated that aberrant expression of ErbB receptors can spontaneously induce immune response by breaking tolerance for these self-antigens in patients with several cancers, including breast, lung, colon, prostate, pancreas, stomach, bladder, liver, testis, and lymphoma (153). However, despite the high level of ErbB receptors expression in HNSCC, it was reported that natural tumor-specific humoral immune responses in HNSCC patients are poor (26). On the other hand, the presence of EGFR-specific CD8^+^ T cells was observed in the circulation of HNSCC patients with high EGFR scores, suggesting that EGFR overexpression on tumor cells can elicit specific T cell responses (154). In this regard, the observation that elevated circulating EGFR-specific T cells are found in HNSCC patients treated with the anti-EGFR antibodies Cetuximab and Nimotuzumab (155, 156), suggests that the presence of specific immune responses against ErbB receptors could be boosted by EGFR-targeted treatments. Actually, the efficacy of Cetuximab appears to involve its ability to trigger antibody-dependent cell-mediated cytotoxicity (ADCC) on NK cells and to promote crosstalk between NK and antigen presenting cells, thereby leading to the generation of EGFR-specific T cells (157). Furthermore, recent evidence obtained in preclinical models of different cancers, including HNSCC, demonstrate that Cetuximab can induce immunogenic cell death, i.e., a type of cell death involving the release of damage-associated molecular patterns (DAMPs) capable to trigger the generation of CD8^+^ T lymphocytes and lead to tumor-specific immunological memory (158, 159).

The therapeutic potential of regimens based on the combination of ICIs and ErbB-targeting agents has been investigated in several studies and many clinical trials are also underway in this regard (160; Table 2). The results of a recent study performed on a small number of PD-L1-positive oral HNSCC patients indicate that the combination of PD-1 inhibitors (Nivolumab or Sintilimab), anti-EGFR targeted therapy (Nimotuzumab) and chemotherapy (Paclitaxel) could improve the response rate and survival outcome (161). In a case report of a patient with recurrent/metastatic oral squamous cell carcinoma, the efficacy of the PD-1 inhibitor Nivolumab in combination with the anti-EGFR antibody Nimotuzumab and radiotherapy has also been described in terms of decreased metabolic activity in cancer cells, reduction of lung lesions dimensions, progression-free survival and tolerable safety profile (162). To mention a few of the ongoing studies, the triple combination of ICIs, Cetuximab and radiotherapy for patients with advanced HNSCC is being investigated in clinical trials employing the anti-PD-L1 antibodies Avelumab and Durvalumab and the anti-CTLA-4 antibody Ipilimumab (Table 2; 157). As regards the Cetuximab/Ipilimumab/radiotherapy combination, preliminary results indicate that the efficacy of this regimen is comparable to that of the standard cisplatin/radiotherapy combination in terms of progression-free and overall survival but offers the advantage of avoiding the administration of the heavily cytotoxic cisplatin (125). The combination of Cetuximab and ICIs such as Pembrolizumab or Avelumab is also investigated in patients with recurrent/metastatic HNSCC (Table 2), and promising clinical activity has been reported for the Cetuximab/Pembrolizumab combination (126). Beside Cetuximab, other ErbB-targeting agents are being evaluated in combination with ICIs for HNSCC, including the anti-ErbB2 antibodies Margetuximab, BDC-1001 and SBT6050, and the EGFR/ErbB2/ErbB4 inhibitor Afatinib (Table 2).

In the search for increasingly effective immunotherapeutic strategies for HNSCC, other approaches are also being investigated, such as those based on chimeric antigen receptor (CAR)-immune cell therapy, oncolytic virus therapy, and vaccines (160). In this regard, CAR-immune cell therapies are under evaluation in HNSCC patients, in trials based on the transfer of different types of immune cells armed with chimeric receptors able to target one or multiple ErbBs. One of such trials is underway to test the safety of the intratumoral administration of autologous T-cells engineered to express a second generation CAR able to engage multiple ErbB dimers that are commonly upregulated in HNSCC (163; Table 2). Trials based on the adoptive transfer of immune cells engineered to target ErbB2, such as NK cells, macrophages or T cells, are also underway for patients with ErbB2-expressing tumors, including HNSCC (Table 2).

As a final remark, the clinical evidence accumulated so far indicates that the response to immunotherapy in HNSCC patients appears on the whole variable (160). This evidence highlights the urge to identify biomarkers able to predict clinical responses in order to select the best therapeutic regimen for personalized, tailored treatments.

## 7. Conclusion

While the incidence of HNSCC is raising worldwide, overall outcomes remain poor due to the limited tumor responsivity to radiation and drug regimens, leading to treatment failure, relapses and disease progression, thus highlighting the urge for novel, more effective therapeutic strategies. Although the EGFR has long been recognized as an established oncogene and a therapeutic target, the full potential of targeting this receptor and the related ErbBs in HNSCC is yet to be fully explored. For instance, although most studies have been focused on the involvement of the EGFR in HNSCCs, the available data indicate that a good proportion of these tumors may simultaneously express multiple ErbB receptors. In this regard, the impact of ErbB receptors cooperative signaling on HNSCC pathogenesis, outcomes and therapeutic responses is an issue which will deserve further investigation. Another open field of research is based on the evidence that non-canonical ErbB signaling mechanisms are involved in HNSCC progression and resistance to therapy, which may lead to the development of therapeutic agents aimed at targeting EGFR nuclear translocation or EGFR/ErbB2 exosomal transfer. Future studies in these fields may also provide new important findings on the involvement of canonical and non-canonical ErbB signaling in the modulation of the tumor microenvironment, useful for the optimization of therapeutic strategies based on the combination of ErbB-targeting agents and immunotherapy approaches.

## Author contributions

CP, MB, CF, LA, LC, AR, DN, VA, and AB were responsible for conceptualization, methodology, formal analysis, writing, figure, and original draft preparation. LM and RB were responsible for conceptualization, review and editing and for funding acquisition. All authors contributed to the article and approved the submitted version.

## Funding

This study was funded by grants from Ministero dell’Università e della Ricerca, PRIN 2020 (BeiR20Prin, CUP: E85F22000060006 to RB), and by a grant from the University of Rome “Sapienza,” Ateneo 2019 (#RM11916B74788236 to LM).

## Conflict of interest

The authors declare that the research was conducted in the absence of any commercial or financial relationships that could be construed as a potential conflict of interest.

## Publisher’s note

All claims expressed in this article are solely those of the authors and do not necessarily represent those of their affiliated organizations, or those of the publisher, the editors and the reviewers. Any product that may be evaluated in this article, or claim that may be made by its manufacturer, is not guaranteed or endorsed by the publisher.

## Footnotes

^1^ https://www.cancer.gov/types/head-and-neck/head-neck-fact-sheet#r1

^2^ tcpaportal.org
